# Gradient Expansions for the Large-Coupling Strength Limit of the Møller–Plesset Adiabatic Connection

**DOI:** 10.1021/acs.jctc.1c01206

**Published:** 2022-02-18

**Authors:** Timothy J. Daas, Derk P. Kooi, Arthur J. A. F. Grooteman, Michael Seidl, Paola Gori-Giorgi

**Affiliations:** Department of Chemistry & Pharmaceutical Sciences and Amsterdam Institute of Molecular and Life Sciences (AIMMS), Faculty of Science, Vrije Universiteit, De Boelelaan 1083, 1081HV Amsterdam, The Netherlands

## Abstract

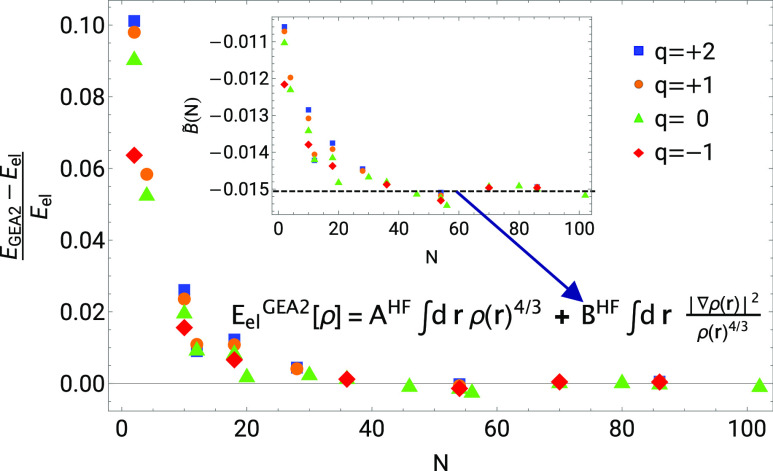

The adiabatic connection that has, as weak-interaction expansion, the Møller–Plesset perturbation series has been recently shown to have a large coupling-strength expansion, in terms of functionals of the Hartree–Fock density with a clear physical meaning. In this work, we accurately evaluate these density functionals and we extract second-order gradient coefficients from the data for neutral atoms, following ideas similar to the ones used in the literature for exchange, with some modifications. These new gradient expansions will be the key ingredient for performing interpolations that have already been shown to reduce dramatically MP2 errors for large noncovalent complexes. As a byproduct, our investigation of neutral atoms with large number of electrons *N* indicates that the second-order gradient expansion for exchange grows as *N* log(*N*) rather than as *N*, as often reported in the literature.

## 1 Introduction

Adiabatic connections (ACs) between an easy-to-solve Hamiltonian and the physical, many-electron one, have always played a crucial role in building approximations in electronic structure theory. In density functional theory (DFT), the standard AC connects the Kohn–Sham (KS) Hamiltonian with the physical one by turning on, via a parameter λ, the electron–electron interaction while keeping the one-electron density ρ(**r**) fixed^1^ (central column of Table 1). In this case, the series expansion of the correlation energy at small coupling strengths (λ → 0) is given by the Görling–Levy (GL) perturbation theory.^2^ In the opposite limit of large-coupling strengths (λ → ∞), the correlation energy is determined by the strictly correlated-electrons (SCE) physics,^3−6^ which yields the leading term. The next order is given by zero-point (ZP) oscillations^7−10^ around the SCE manifold. A possible strategy to build approximations for the correlation energy is to interpolate between these two opposite limits, generalizing to any nonuniform density^7,11−16^ the idea that Wigner^17^ used for jellium. The advantage of such an approach is that it is not biased toward the weakly correlated regime. The lack of size consistency of these interpolations can be easily corrected at zero computational cost.^14^

**Table 1:** Two Adiabatic Connections (ACs) Considered in This Work^a^

	DFT adiabatic connection	Møller–Plesset adiabatic connection
*Ĥ*_λ_	*T̂* + *V̂*_ext_ + λ*V̂*_ee_ + *V̂*_λ_[ρ]	*T̂* + *V̂*_ext_ + *V̂*^HF^ + λ (*V̂*_ee_ – *V̂*^HF^)
		*V̂*^HF^ = *Ĵ*[ρ^HF^] – *K̂*[{ϕ_*i*_^HF^}]
		
ρ_λ=0_	ρ	ρ^HF^
		
ρ_λ=1_	ρ	ρ
		
ρ_λ_	ρ	ρ_λ_
		
*W*_*c*,λ_	⟨Ψ_λ_|*V̂*_ee_|Ψ_λ_⟩ – ⟨Ψ_0_|*V̂*_ee_|Ψ_0_⟩	⟨Ψ_λ_|*V̂*_ee_ – *V̂*^HF^|Ψ_λ_⟩ – ⟨Ψ_0_|*V̂*_ee_ – *V̂*^HF^|Ψ_0_⟩
		
*E*_*c*_	∫_0_^1^*W*_*c*,λ_^DFT^ dλ	∫_0_^1^*W*_*c*,λ_^HF^ dλ
		
*W*_*c*,λ→0_	∑_*n*=2_^∞^*nE*_c_^GLn^λ^*n*–1^	∑_*n*=2_^∞^*nE_c_*^MP^*n*λ^*n*–1^
		
*W*_*c*,λ→∞_	*W*_*c*,∞_^DFT^ + *W*_1/2_^DFT^λ^–1/2^ + *O*(λ^–5/4^)	*W*_*c*,∞_^HF^ + *W*_1/2_^HF^λ^–1/2^ + *W*_3/4_^HF^λ^–3/4^ + *O*(λ^–5/4^)

a Middle column: the standard density-fixed DFT AC that starts at λ = 0 with the Kohn–Sham determinant. Right column: the AC that has the Møller–Plesset series as expansion for small coupling strengths λ and starts at λ = 0 with the Hartree–Fock Slater determinant.

More recently,^18^ the same interpolation idea has been applied to the AC that has the Møller–Plesset (MP) series as perturbation expansion at small coupling strengths λ (right panel of Table 1), connecting the Hartree–Fock (HF) Hamiltonian with the physical one. The λ → ∞ expansion of this MP AC is given by functionals of the Hartree–Fock density ρ^HF^(**r**), with a clear physical meaning.^19,20^ The strong-coupling functionals of the DFT and the MP ACs are essentially electrostatic energies, whose exact evaluation for large particle numbers is demanding, but while for the DFT AC there are rather accurate second-order gradient expansion approximations (GEA2)^4,7,21^ and, more recently, also generalized gradient approximations^16^ (GGA), for the MP AC these approximations are not yet available. For this reason, in a very recent work^18^ the λ → ∞ functionals of the MP AC have been modeled in an empirical way, starting from the GEA2 of the DFT ones. Quite remarkably, interpolations combined with this simple empirical model already provide very accurate results for noncovalent interactions (NCI), reducing the MP2 error by up to a factor 10 in the L7 dataset,^22^ without spoiling MP2 energies for the cases in which they are accurate.^18^ These interpolations work very well for diverse NCI’s such as charge transfer and dipolar interactions, and they are able to correct MP2 both when it overbinds and when it underbinds, as they are able to take into account the change from concave to convex behavior of the MP AC.^18^ Their appealing feature is that they use 100% of HF exchange and MP2 correlation energy, and it is the interpolation that decides for each system how much to correct with respect to MP2. This way, dispersion corrections are not needed at all to get accurate NCIs.^18^

The purpose of this work is to derive the missing GEA2 for the strong-coupling functionals of the MP AC, in order to reduce empiricism and hopefully increase the accuracy of the interpolations along the MP AC. To this purpose, we use the ideas derived from the semiclassical limit of neutral atoms, which have been used in recent years in DFT for the analysis of the exchange and correlation functionals,^23−28^ yielding new approximations such as PBEsol.^29^ As we shall see, the functionals we need to approximate allow us to probe these ideas more extensively, revealing several interesting features that could be used more generally to build DFT approximations. We also notice that an additional term with respect to refs^23, 24, 29^ should be present in the second-order gradient expansion for exchange.

The paper is organized as follows. In section 2, we quickly review the large-coupling-strength functionals of the MP AC, discussing their physical meaning and the crucial differences with those of the DFT AC. Then, in section 3, we focus on the gradient expansion of the leading term at large coupling strengths: we perform an extensive analysis by filling more and more particles in a given density profile, and also by considering closed-shell neutral atoms and ions, up to the Bohr atom densities, which provide the limit of highly ionized atoms. We compute the functional in a numerically accurate way and determine a second-order gradient coefficient for the neutral-atoms case. We also discuss differences with the work of refs (23 and 24), providing an analysis that should also be relevant for the exchange and correlation functionals of DFT. In section 4, along similar lines, we extract the GEA2 coefficient for the next leading term of the MP AC large-coupling-strength expansion. The computational details are described in section 5, and the last section (section6) is devoted to conclusions and perspectives. More technical details, a curious behavior of *N* = 2 ions, and the discussion of an electrostatic model similar to the one used to derive the GEA2 coefficient of DFT are reported in the Appendix. Hartree atomic units will be used throughout this work.

## 2 The Large Coupling Strength Functionals of the Møller–Plesset Adiabatic Connection

### 2.1 The Møller–Plesset Adiabatic Connection

To start, we must introduce the Møller–Plesset Adiabatic Connection (MP AC), which has the following Hamiltonian:
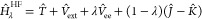
1with *T̂* the kinetic energy, *V̂*_ee_ the electron–electron repulsion, and *V̂*_ext_ the external potential due to the nuclei. The operators *Ĵ* = *Ĵ*[ρ^HF^] and *K̂* = *K̂*[{ϕ_*i*_^HF^}] are the standard Hartree–Fock (HF) Coulomb and exchange operators in terms of the HF density ρ^HF^ and the corresponding occupied orbitals ϕ_*i*_^HF^, respectively, which are determined in the initial HF calculation and are not dependent on λ. Notice that, in our notation, *K̂* is positive definite. This Hamiltonian links the Hartree–Fock system (λ = 0) to the physical system (λ = 1). The HF (or standard wave function theory) correlation energy, using the Hellmann–Feynman theorem, is given by
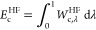
2with *W*_*c*,λ_^HF^ being the MP AC integrand,
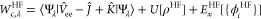
3and Ψ_λ_ the wave function that minimizes the expectation value of the Hamiltonian of eq 1. The last two terms, *U*[ρ^HF^] and *E*_*x*_[{ϕ_*i*_^HF^}], are the Hartree energy and the HF exchange energy, respectively, whose sum gives minus the expectation value of *V̂*_*ee*_ – *Ĵ* + *K̂* on the HF Slater determinant (see right column of Table 1). The small-λ expansion of *W*_*c*,λ_^HF^ is the familiar MP perturbation series,
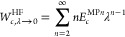
4

### 2.2 The λ → ∞ Expansion of the Møller–Plesset Adiabatic Connection

The large-λ expansion of the MP AC has recently been uncovered^19,20^ for closed-shell systems as follows:
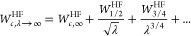
5
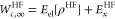
6
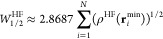
7
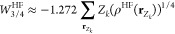
8The leading order, eq 6, contains the electrostatic-energy density functional *E*_el_[ρ], which entails a classical electrostatic minimization,
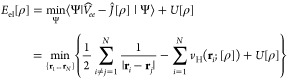
9with
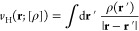
10and
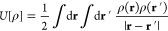
11The density functional *E*_el_[ρ] can be understood as the total electrostatic energy of a distribution of *N* negative point charges and continuous “positive” charges with density ρ(**r**). In other words, the λ → ∞ limit of the MP AC is a crystal of classical electrons bound by minus the Hartree potential generated by the HF density.^19,20^ The resulting minimizing positions {**r**_1_^min^... **r**_*N*_^min^} in eq 9, in turn, determine the next leading term, for which eq 7 provides a rigorous variational estimate for closed-shell systems.^20^ This term is given by zero-point oscillations around the minimizing positions enhanced by the exchange operator *K̂*, which mixes in excited harmonic oscillator states.^20^ Finally, the sum in eq 8 only runs over those minimizing positions of eq 9 that happen to be at a nucleus, and it is also a variational estimate.^20^ These first three leading terms provide a rigorous framework to link MP perturbation theory with DFT, in terms of functionals of the HF density. In practice, we do not want to perform each time the classical minimization of eq 9, which is known to have many local minima and whose cost increases rapidly with *N*. We rather wish to find good gradient expansion approximations for the first two terms in the expansion described by eq 5. The third term, *W*_3/4_^HF^ of eq 8, could instead be approximated by making the assumption that, in a large system, there is one minimizing position at each nucleus, transforming it into a functional of the HF density at the nuclei.

### 2.3 Comparison with the λ → ∞ Expansion of the DFT AC

In a recent work where an interpolation for *W*_*c*,λ_^HF^ between MP2 and the λ → ∞ limit has been built and tested,^18^ the functional *W*_*c*,∞_^HF^ of eq 6 has been approximated in terms of the strong interaction limit of the DFT AC, using the following inequality:^19^
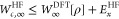
12The DFT AC of the central column of Table 1 uses the Hamiltonian,
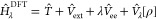
13with *V̂*_λ_[ρ] = ∑_*i*=1_^*N*^*v*_λ_(**r**_*i*_, [ρ]) being the one-body potential that forces the density to be equal to the physical one for all values of λ. With this Hamiltonian, the KS exchange-correlation (XC) energy is given by
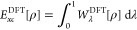
14where the DFT coupling constant integrand is
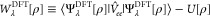
15and Ψ_λ_^DFT^[ρ] is the wave function that minimizes the expectation value of eq 13. Although the λ → ∞ expansion of the DFT AC has a similar form as the MP AC one of eq 5, there are important differences between the two. The first one is the lack of the λ^–3/4^ term in the DFT AC, which has the following large-coupling expansion:^4,7^
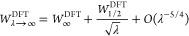
16The reason why the MP AC can have a λ^–3/4^ term is that in this case there is no constraint on the density, and the electrons thus localize around the minimizing positions {**r**_1_^min^... **r**_*N*_^min^}. The density approaches asymptotically, as λ → ∞, a sum of Dirac delta functions centered around these minimizing positions. If one of the **r**_*i*_^min^ happens to be at a nucleus, the nonanalyticity of the Coulomb nuclear attraction and of the cusp in the HF orbitals and density give rise to this term.^20^

In the DFT AC case, the density constraint enforces Ψ_∞_^DFT^ to be a superposition of infinitely many classical configurations,^4^ so the one with an electron at a nucleus has infinitesimal weight.

The inequality described by eq 12 can be understood on simple physical terms: the functional *W*_∞_^DFT^[ρ] can be reformulated as^21^
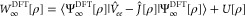
17where we have simply used the fact that the expectation of *Ĵ*[ρ] on any wave function with density ρ(**r**) is 2*U*[ρ]. Then, we can interpret^21^*W*_∞_^DFT^[ρ] as the electrostatic energy of a system of classical electrons forced to have density ρ immersed in a classical background of charge density ρ of opposite sign. Notice that Ψ_∞_^DFT^[ρ] *does not minimize* this electrostatic energy, but it is given by
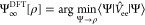
18The functional *E*_el_[ρ] of eq 9, in contrast, is obtained by letting Ψ relax to its minimum in eq 17, which directly implies
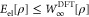
19Adding *E*_*x*_^HF^ to both sides of this inequality yields eq 12.

### 2.4 Semilocal Functionals for the λ → ∞ Expansion of the DFT AC

In ref (18). parameters were added to both terms on the right-hand-side of eq 12 to be fitted to the S22 dataset.^30,31^ This inequality was used due to the lack of approximations for *E*_el_[ρ], but also to allow the functional to be more flexible to approximate the missing but very large second-order term. Although the exact evaluation of *W*_∞_^DFT^[ρ] is even more expensive than the one of *W*_∞_^HF^[ρ], an inexpensive^21^ but accurate^4,7^ approximation called the Point Charge Plus Continuum (PC) model exists, which is a GEA2 functional:
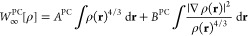
20with *A*^PC^ =  and *B*^PC^ =  ≈ 0.005317. The PC model was built from the physical interpretation of *W*_∞_^DFT^[ρ] provided by eq 17: perfectly correlated electrons that need to minimize their interaction while giving the same density ρ of the classical positive background will tend to *neutralize* the classical charge distribution ρ (which is different than *minimize* the total electrostatic energy, as in *E*_el_[ρ]). Along similar lines, by considering zero-point oscillations around the PC positions, a GEA2 functional for the second-order term, was constructed:^21^
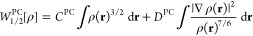
21with *C*^PC^ = 1/2(3π)^1/2^ ≈ 1.535 and, from ref (7), *D*^PC^ = −0.028957, where *D*^PC^ is fixed to reproduce the helium-atom exact result.^7^ In newer work by Constantin,^16^ GGA functionals for both terms were derived to fix, among other things, the diverging asymptotics of the XC potentials.^32^ However, these GGA’s have larger errors than the original PC model when compared with accurate SCE values for small atoms. Notice that, in contrast to the DFT AC (where self-consistent calculations should in principle be carried out), we here do not need the functional derivatives of these quantities, as the MP AC is designed to directly give the HF correlation energy in a post-self-consistent-field manner.

## 3 Second-Order Gradient Expansion for *E*_el_[ρ]

In this section, we wish to derive a gradient expansion for *E*_el_[ρ] of eq 9. As detailed in Appendix C, we cannot proceed along lines similar to the derivation of the PC model used for the DFT AC, because the charge distribution with the electrostatic energy *E*_el_[ρ] cannot easily be divided into weakly interacting cells. Moreover, we are only interested in *E*_el_[ρ] for ρ(**r**) that are HF densities of atoms and molecules. For this reason, we follow the procedure used for the DFT exchange functional *E*_*x*_^DFT^[ρ] in refs (23 and 24) with some modifications. This procedure extracts the GEA2 coefficient from accurate data, and is very suitable because, under uniform coordinate scaling at fixed particle number *N*,
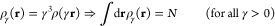
22our functional *E*_el_[ρ] displays the same scaling behavior as exchange,
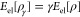
23since *v*_H_([ρ_γ_], **r**) = γ*v*_H_([ρ], γ**r**) and *U*[ρ_γ_] = γ*U*[ρ].

In practice, we wish to determine whether, for slowly varying densities, *E*_el_[ρ] is well-approximated by a second-order gradient expansion:
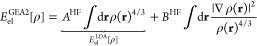
24The powers ρ(**r**)^4/3^ in the two terms of this expression are a necessary consequence of the exact scaling law of eq 23. Defining the usual reduced gradient *x* of the density ρ,
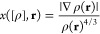
25which essentially gives the relative change of the density on the scale of the average interparticle distance, eq 24 can also be written as
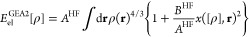
26The GEA2 expression should become more and more accurate as *x* → 0, and our goal is to determine the values of *A*^HF^ and *B*^HF^. As we will discuss later, while *A*^HF^ is universal, the coefficient *B*^HF^ seems to be dependent on how the slowly varying limit is approached, similarly to what happens with the DFT exchange functional.^23,27^

### 3.1 LDA Coefficient *A*^HF^

The uniform density limit *N* → ∞ of a constant droplet density  with radius *R*_*N*_ = *N*^1/3^*r*_*s*_, taken per particle:
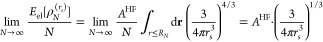
27is equivalent to the jellium case^33^ and has been analyzed already in ref (20). The result is the Wigner crystal energy per particle,^34^, leading to
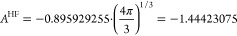
28Notice that *A*^HF^ = *A*^DFT^, where the latter is slightly different than the PC value *A*^PC^, which replaces 0.8959 ... with 0.9. Note the rigorous proof in refs (33 and 35) that *A*^DFT^ is in fact exactly given by the Wigner crystal.

### 3.2 Particle-Number Scalings

As discussed in refs (23 and 24), the slowly varying limit can be approached in different ways. An extended system with uniform density can be perturbed with a slowly varying density distortion, but the resulting GEA2 coefficient might not be the one useful for chemistry.^23^ More generally^23,36^ for any functional that scales as eq 23, we can reach the slowly varying limit by simply putting more and more electrons in a density profile ρ̅(**r**) with ∫d**r** ρ̅(**r**) = 1, by generating a discrete sequence of densities with increasing particle numbers *N* = 1, 2, 3, ..., using the scaling^36^
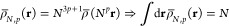
29With growing *N*, for all these densities the reduced gradient of eq 25 becomes increasingly weak,
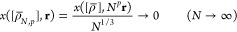
30provided that
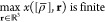
31Examples of relevant values of *p* are

- *p* = 1/3: the Thomas–Fermi scaling of neutral atoms^23,28,37^*N* = *Z*;
- *p* = −2/3: the Thomas–Fermi scaling of the Bohr atoms;^27,28,38,39^
- *p* = 0: the scaling used in refs (40−42) to analyze the Lieb–Oxford bound;
- *p* = −1/3: the scaling used in ref (43) to analyze the asymptotic exactness of the local density approximation.

For any density functional *G*[ρ] that, under the uniform coordinate scaling of eq 22, behaves as
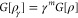
32for a fixed profile ρ̅, all the different choices of *p* in eq 29 are equivalent and simply related to the case *p* = 0,
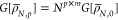
33For functionals that do not display a simple scaling behavior, like correlation in DFT, different values of *p* lead to different interesting regimes, as discussed in refs (23 and 36).

**Figure 1:**
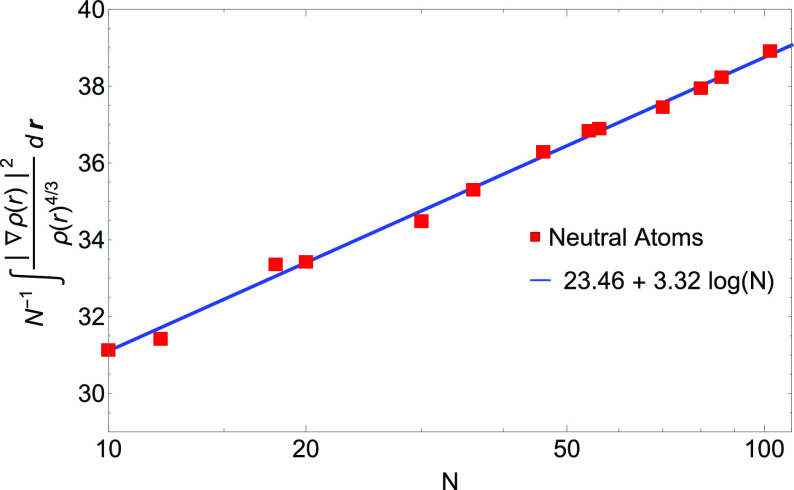
GEA2 integral of eq 36 for the Hartree–Fock densities of neutral atoms, *I*_GEA2_[ρ_*N*=*Z*_], divided by the number of electrons *N* (log scale on the *x*-axis). Numerical values (red dots) are compared with a logarithmic fit (blue line).

When *N* grows, because of eq 30, we expect the gradient expansion of eq 24 to become more and more accurate. Then, by inserting the density ρ̅_*N,p*_ into eq 24, one gets the following large-*N* expansion
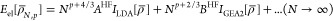
34where
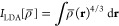
35
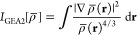
36Clearly, eq 34 holds only as long as the integrals *I*_LDA_[ρ̅] and *I*_GEA2_[ρ̅] are finite for the given density profile ρ̅. In refs (23, 24) the fact that the neutral atoms densities for large *N* asymptotically satisfy the Thomas–Fermi (TF) scaling with *p* = 1/3,
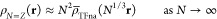
37with ρ̅_TFna_(**r**) the TF profile (integrating to 1) for neutral atoms,^25−28,37^ led to the conclusion that their exchange energy, as a function of *N* = *Z* should have, to leading orders, the large-*N* expansion *a*_*x*_*N*^5/3^+*b*_*x*_*N*. Extracting *b*_*x*_ from exchange energies of neutral atoms allowed to fix the GEA2 coefficient for exchange in ref (24). However, while the GEA2 integral *I*_GEA2_[ρ_*N*=*Z*_] for neutral atoms is finite, the integral *I*_GEA2_[ρ̅_TFna_] for the asymptotic TF profile diverges (while *I*_LDA_[ρ̅_TFna_] is also finite). This does not automatically imply that *I*_GEA2_[ρ_*N*=*Z*_] should not increase linearly with *N*, as expected from eq 34 with *p* = 1/3, since TF theory should not give exact information at this order. Nonetheless, we find numerical evidence (see Figure 1) that the GEA2 integral *I*_GEA2_[ρ_*N* = *Z*_^HF^] for Hartree–Fock densities of neutral atoms increases as *N* log(*N*) rather than as *N*.

A case for which it is even simpler to make a detailed numerical analysis of *I*_GEA2_ is the Bohr atoms,^27,28,38^ which have densities constructed by occupying hydrogenic orbitals:
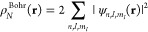
38and can be considered^27,38^ as a limiting case for ions with *Z* ≫ *N*. The latter have densities that, as *Z* → ∞, approach those of the Bohr atom scaled as in eq 22 with γ = *Z*,
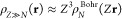
39As *N* → ∞, the densities ρ_*N*_^Bohr^(**r**) of eq 38 approach the Bohr atom TF profile^27,28,38^ ρ̅_TFBohr_ with *p* = −2/3,
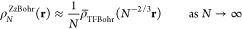
40Again, *I*_LDA_[ρ̅_TFBohr_] is finite while *I*_GEA2_[ρ̅_TFBohr_] diverges.

**Figure 2:**
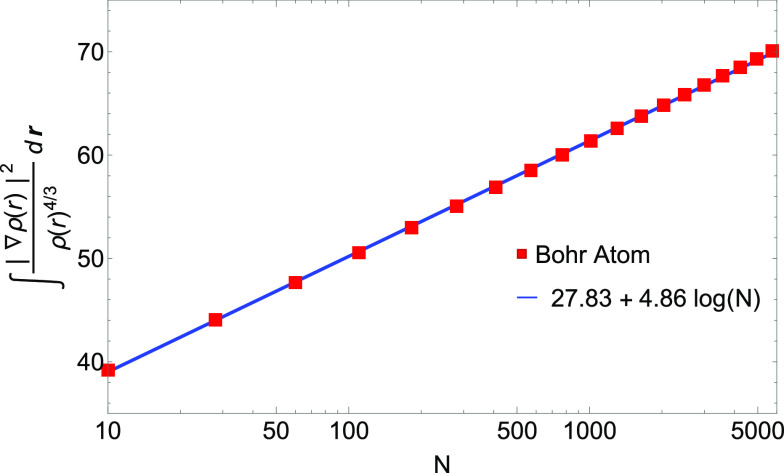
GEA2 integral of eq 36 for the Bohr atom densities, *I*_GEA2_[ρ_*N*_^Bohr^] (log scale on the *x*-axis). Numerical values (red dots) are compared with a logarithmic fit (blue line).

If eq 34 would hold with *p* = −2/3, *I*_GEA2_[ρ_*N*_^Bohr^] should have a tendency toward a constant when *N* → ∞. Instead, we clearly see (Figure 2) that it grows as log(*N*). For this case, everything is analytic and it is easy to reach very large *N*, evaluating the GEA2 integral to high accuracy.

A detailed derivation of the behavior of *I*_GEA2_[ρ], as a function of *N* for neutral atoms and for Bohr atoms, confirming the numerical evidence reported here, is also being performed independently by Argaman et al.^44^

### 3.3 Extracting the GEA2 Coefficient *B*^HF^

**Figure 3:**
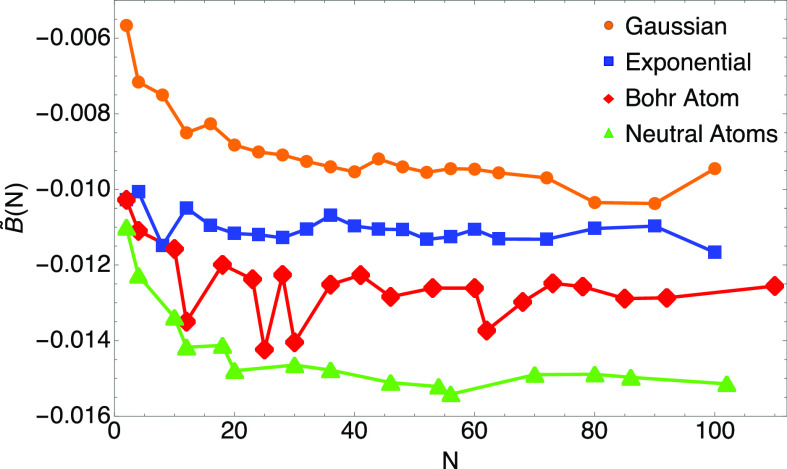
Plot of eq 41 for the four cases described in section 3.3.

The analysis in the previous section suggests that extraction of the GEA2 coefficient should not be done by using values of *E*_el_[ρ], as a function of *N* and fitting coefficients from eq 34, as this seems to be safe only for a scaled known profile (as in eq 29), but not for atomic densities. For this reason, we follow a route slightly different than the one used for exchange in ref (24). Namely, we directly compute
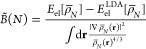
41The idea is that if a GEA2 expansion for *E*_el_[ρ] exists, we should observe that *B̃*(*N* → ∞) tends to a constant, which will be the sought *B*^HF^. However, such constant might not be the same for different profiles ρ̅ or when we use the neutral atoms or the Bohr atom densities. Indeed, this seems to be the case: in Figure 3, we show for different particle numbers *N*:

(1) Our numerical values *B̃*(*N*) for the exponential profile
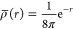
42
(2) Our numerical values *B̃*(*N*) for the Gaussian profile
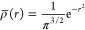
43
(3) Our numerical values *B̃*(*N*) for the Hartree–Fock densities ρ_*N* = *Z*_^HF^(**r**) of neutral atoms.
(4) Our numerical values *B̃*(*N*) for the Bohr atom densities ρ_*N*_^Bohr^(**r**) of eq 38, including some cases in which we did not completely fill all the  values for a given principal quantum number *n*. Notice that these latter cases cannot always be seen as the limit of highly ionized atoms, as degeneracy must be taken into account more carefully.

The computational details behind the evaluation of *E*_el_[ρ] for each case are described in section 5. We see that these four sequences of data for *B̃*(*N*) seem to approach four different limits as *N* grows. Regarding the Bohr atoms, the cases for which the value of *B̃*(*N*) suddenly decreases to a value much closer to the one of neutral atoms are those in which we added an extra pair of *s* electrons to a completely filled shell. For example, *N* = 12 is obtained by adding 3*s*^2^ to the filled *n* = 2 shell, and similarly for *N* = 30 and *N* = 62. The case *N* = 25 is realized by filling the orbitals as in the Mn atom. From Figure 3 we can conclude that there exists no unique GEA2 and that we should choose one of these *B*^HF^’s for our new GEA2 functional. As for the case of the exchange functional,^23,24^ the most useful value for chemistry should be the one of neutral atoms.

**Figure 4:**
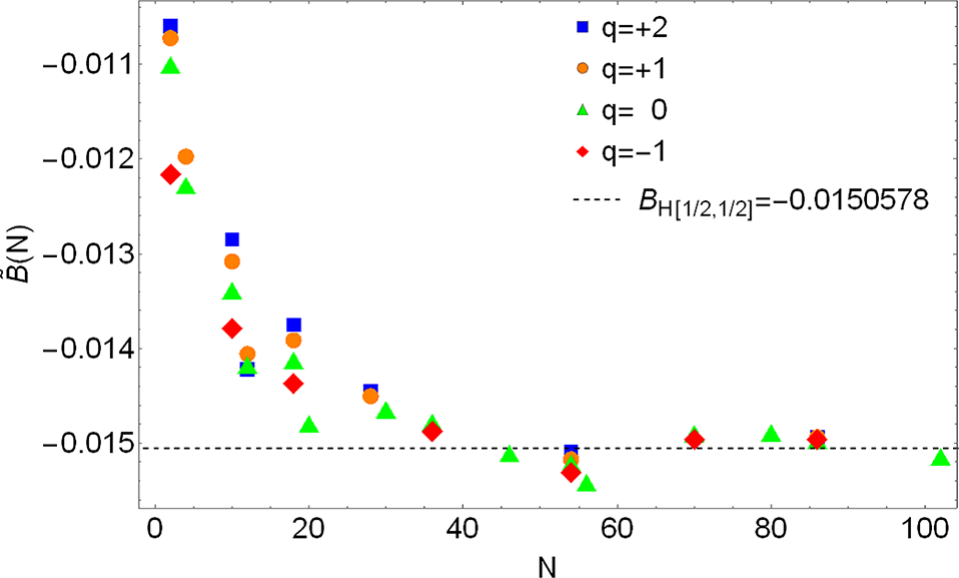
Value *B*_H[^1^/_2_, ^1^/_2_]_^HF^ = −0.0150578 that makes the GEA2 exact for the spin-unpolarized H atom accurately recovers the large *N* limit of *B̃*(*N*) of eq 41 for closed-shell neutral atoms (*q* = 0) and slightly charged closed shell ions, with *q* = +1, +2, and −1.

**Figure 5:**
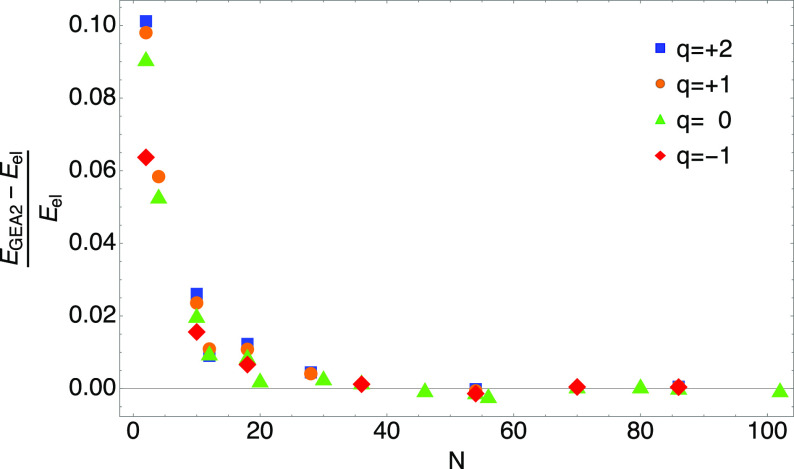
Relative error of the GEA2 expansion for the functional *E*_el_[ρ] with *B*^HF^ = *B*_H_^HF[^1^/_2_, ^1^/_2_]^ = −0.0150578 for closed-shell neutral atoms (*q* = 0) and ions with *q* = +1, +2, and −1.

We noticed that if we fix *B*^HF^ to make the GEA2 exact for the spin-unpolarized H atom^20^ (with 1/2 spin-up and 1/2 spin-down electrons^45^),
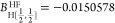
44we recover the large *N* limit of closed-shell neutral atoms and closed-shell ions with charges +1, +2, and −1 quite closely, as shown in Figure 4. We thus fix the GEA2 coefficient *B*^HF^ to this value, which seems to be as good as a fitted one, although we lack at this point a theoretical justification of why the H[^1^/_2_, ^1^/_2_] should provide such a good number. In Figure 5, we show the relative error of the GEA2 expansion, which, as expected, goes to zero for large neutral atoms and slightly charged ions.

**Figure 6:**
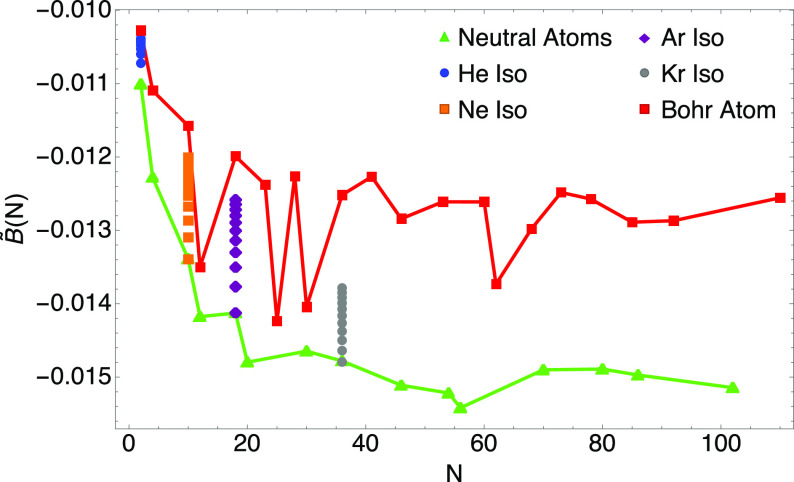
*B̃*(*N*) of eq 41 for neutral atoms, Bohr atoms and for selected noble-gas isoelectronic series. We see how *B̃*(*N*) goes from one limit to the other as the nuclear charge *Z* is increased at fixed *N*.

However, we should stress that the GEA2 with *B*^HF^ of eq 44 misses the other^27^ slowly varying limit of Bohr atoms with large *N*, which can be regarded as the limit^27^*Z* ≫ *N* ≫ 1. To better illustrate the issue, we show in Figure 6 the values *B̃*(*N*) only for the closed-shell neutral atoms, the Bohr atoms and for selected noble-gas isoelectronic series: we then see how *B̃*(*N*) goes from one limit to the other as the nuclear charge *Z* is increased at a fixed electron number *N*. An approximation able to cover this entire range of values could possibly be designed as a metaGGA, which is a route that will be investigated in future work.

## 4 Second-Order Gradient Expansion for *W*_1/2_^HF^[ρ]

Once the minimization to obtain *E*_el_[ρ] is performed, we automatically get the functional *W*_1/2_^HF^[ρ] of eq 7 by evaluating the HF density in the minimizing positions **r**_*i*_^min^. We should still stress that, while the leading term of eq 6 is exact, eq 7 is only a variational estimate valid for closed-shell systems within restricted HF.^20^ Nevertheless, we can repeat the analysis of the previous section to obtain a GEA2, which, because of the fact that *W*_1/2_^HF^[ρ] satisfies eq 32 with *m* = 3/2, must have the same form as the one for the DFT case of eq 21:
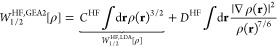
45

### 4.1 LDA Coefficient *C*^HF^

Within the variational expression of eq 7, the LDA coefficient *C*^HF^ is readily evaluated^20^ and equal to 2.8687. Notice that this is not the exact value for a uniform HF density, which should be evaluated by computing the normal modes around the bcc positions in the Wigner crystal and minimizing the total energy in the presence of the nonlocal operator *K̂*, which will mix in excited modes. This analysis, using the techniques recently introduced by Alves et al.,^34^ is the object of a work in progress.

### 4.2 Extraction of the GEA2 Coefficient *D*^HF^

**Figure 7:**
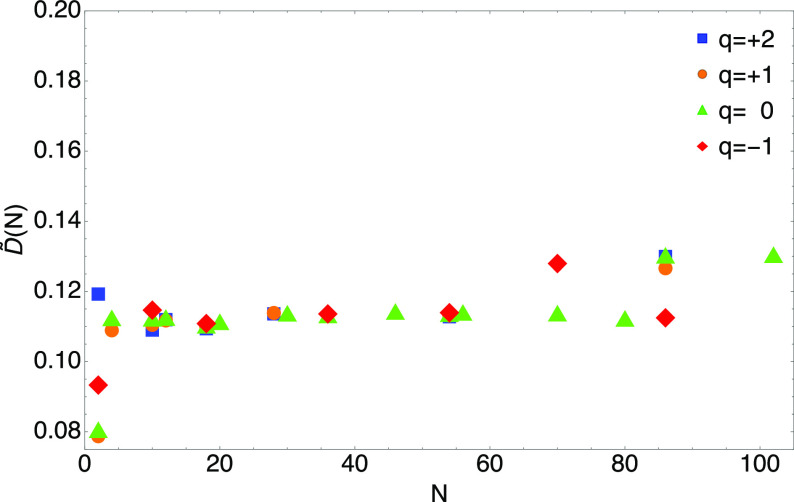
Values of *D̃*(*N*) of eq 46 for closed-shell neutral atoms (*q* = 0) and slightly charged closed shell ions, with *q* = +1, +2, and −1.

We focus only on the relevant case of closed-shell neutral atoms and slightly charged ions, and, in analogy with eq 41, we compute and analyze the function
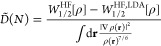
46The results are shown in Figure 7, where we see that *D̃*(*N*) gets rather flat already at *N* ≳ 30 around the value of ∼0.11. However, we also see a step to a slightly higher value, ∼0.13, for the largest *N*. We do not know whether this step is really there or whether it is due to the numerical minimization being trapped in a local minimum. The issue is that, as *N* increases, there are many local minima with very close values of *E*_el_[ρ], which therefore remains rather insensitive if the true global minimum is not reached. However, the functional *W*_1/2_^HF^[ρ] is dependent on the minimizing configuration and it changes more from one local minimum to another. We illustrate this in Appendix B for the case *N* = 2, which undergoes a transition from a symmetric to an asymmetric minimum as the nuclear charge *Z* varies from 2 to 1. From the data of Figure 7, we can get a rough estimate *D*^HF^ ≈ 0.12 ± 0.01.

## 5 Computational Details

To obtain reference values for *E*_el_[ρ] for closed-shell neutral atoms and slightly charged ions, we first performed RHF calculations with PySCF 1.7.6,^46^ with the basis sets specified in Appendix A. For a given set of positions {**r**_1_, **r**_2_, ..., **r**_*N*_}, we calculated the value of *V*_el_, where
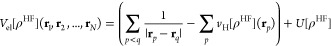
47We computed the value of *v*_H_[ρ^HF^](**r**_*p*_) by contracting,
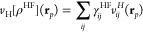
48where γ^HF^ is the Hartree–Fock 1-body Reduced Density Matrix (1-RDM) and the matrix element is given by
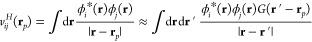
49where a very sharply peaked Gaussian *G* was used to approximate the point charge, which allows for a more efficient computation of the matrix elements using PySCF. To allow for minimization using a quasi-Newton method, we also obtained the gradient of the Hartree potential,
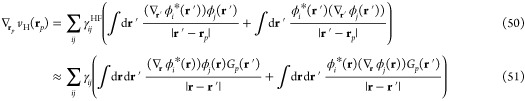
The total gradient then is
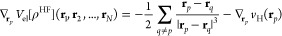
52Finally, *E*_el_[ρ^HF^] was obtained by minimizing *V*_el_[ρ^HF^] using the Broyden–Fletcher–Goldfarb–Shanno (BFGS) algorithm^47−50^ as in the scipy.optimize.minimize function of scipy.^51^

For selected cases, such as Ne and Ar, we have also double-checked the minimum by using Mathematica 12.3.1, experimenting with different minimizers. For the scaled densities and the Bohr atoms, we have used both Mathematica and Python with the same scipy.optimize.minimize function used for the HF densities.

## 6 Conclusions and Perspectives

We have built second-order gradient expansions for the functionals of the large-coupling-strength limit (see last line of the right column of Table 1) of the adiabatic connection that has the Møller–Plesset perturbation series as small-coupling-strength expansion (see eqs 24 and 45). For this purpose, we have used ideas from the literature based on the semiclassical limit of neutral and highly ionized atoms.^23,24,28^ During our study, we have also found numerical evidence (see section 3.2 and Figures 1 and 2) that suggests that the way this semiclassical limit has been used to extract second-order gradient coefficients for exchange should be revised.^23,24,29^

In future work, we will design and test new formulas for the adiabatic connection (AC) of the right-hand side of Table 1 that interpolate between MP2 and these new semilocal functionals at large coupling, including the term proportional to λ^–3/4^, which can be approximated as a functional of the HF density at the nuclei. Previous work^18^ showed that such functionals can be very accurate for noncovalent interactions, correcting the MP2 error for relatively large systems without using dispersion corrections. We will also analyze in the same way the functionals at strong coupling of the DFT AC (last line of the central column of Table 1), although, in this case, obtaining accurate results for large neutral atoms is numerically challenging.

## Figures and Tables

**Figure 8:**
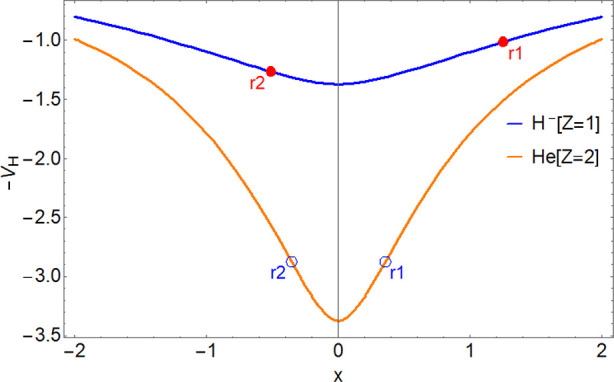
Minus the Hartree potential of the HF densities of He and H^–^, together with the electronic positions that minimize *E*_el_[ρ] (in Hartree atomic units).

**Figure 9:**
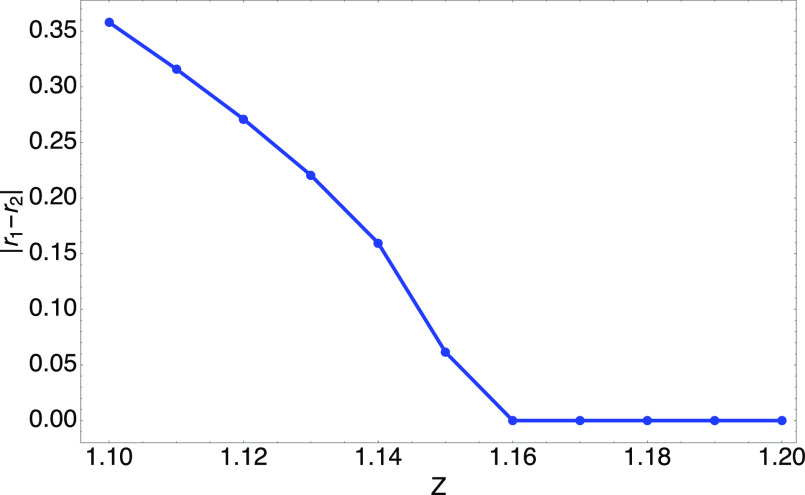
Difference between the distances from the nucleus of the two minimizing positions (Hartree atomic units) for *E*_el_[ρ] for HF densities of ions with *N* = 2 and varying nuclear charge *Z*.

## Appendix A: Basis Sets

For He–Zn^2+^ we used aug-cc-pVQZ basis sets from ref (52). For the heavier atoms ranging from Zn to Xe, we used Jorge AQZP,^53,54^ except for Br^–^, where we used a standard aug-cc-pVQZ basis set. We used Jorge ATZP for Cs to No, except for Ba and Ba^2+^, for which we used the Jorge TZP basis set. For the helium iso-electronic series, we used an aug-cc-pV6Z specifically designed for helium,^55^ whereas for the iso-electronic series of neon and argon we used a standard aug-cc-pV5Z basisset. For the krypton iso-electronic series, we used an aug-cc-pVQZ basis set instead.

## Appendix B: Symmetry Breaking in *N* = 2 Ions

Here, we report a curiosity that we have observed about the minimizing positions of eq 9. Naively, one would expect the minimizing positions for a *N* = 2 atom to be symmetrically distributed on both sides of the well created by −*v*_H_(*r*), which is the case for the helium atom (orange curve) in Figure 8, with minimizing positions in blue. However, when the nuclear charge *Z* is decreased to 1 (H^–^), the minimizing positions (red dots) are now asymmetrically distributed. In Figure 9, we show the difference between the distances from the nucleus of the two minimizing positions as *Z* varies: we see that the change from a symmetric minimum to an asymmetric minimum happens at *Z* = 1.16. In the case of H^–^, a symmetric minimum is still present, but it is only a local one. In Table 2, we report the values of *E*_el_[ρ] for H^–^ for the asymmetric global minimum and the symmetric local one, showing that the difference is small (<0.1%), and any semilocal approximation for *E*_el_[ρ] would have already larger errors than the difference between these two minima. However, the value of *W*_1/2_^HF^[ρ], also reported in the table, does change more significantly for the different minima (∼3%), because it depends on the density at the minimizing positions directly (see eq 7).

**Table 2:** Distances from the Nucleus of the Minimizing Positions *r*_1_ and *r*_2_ (Hartree atomic units), *E*_el_[ρ] and *W*_1/2_^HF^[ρ] of the Symmetric Local Minimum and Asymmetric Global Minimum of H^–^

	H^–^[Sym]	H^–^[Asym]
*r*_1_	0.8477	1.2515
*r*_2_	0.8477	0.5116
*E*_el_[ρ]	–0.9219	–0.9228
*W*_1/2_^HF^[ρ]	1.4545	1.5003

## Appendix C: PC Model for *E*_el_[ρ] and Its Limitations

The PC model^21^ for *W*_∞_^DFT^[ρ] (eq 20) is built by starting from eq 17 for a uniform density, in which the Wigner crystal of strictly correlated electrons is approximated by having each electron surrounded by a sphere of background density exactly integrating to 1 (PC cell). Such an approximation amounts to replace 0.9 with the value of the bcc Madelung constant 0.8959 of eq 28. The GEA2 coefficient *B*^PC^ is then derived by applying a small gradient Γ = |∇ρ(**r**)| by requiring that the PC cell plus its electron have exactly zero dipole moment, a new modified PC cell is constructed, whose center and size are slightly changed. This way, a given density can be thought of as being composed by cells (the PC cells plus their electron) that are weakly interacting, and the total energy can be obtained as a sum of the electrostatic energy of each cell (equal to the background-background interaction plus the electron-background interaction).

In the case of *E*_el_[ρ], instead, we want to minimize the total electrostatic energy. When the density is uniform, there is no other choice than creating the Wigner crystal bcc arrangement, which could be again approximated with PC spherical cells integrating to 1. Now we apply a small gradient and, in analogy with the DFT PC model, we focus on the energy of only one cell. The electron will move from the center of the sphere in the position that minimizes the electrostatic energy, i.e., the minimum of the Hartree potential of the PC cell with dipole. An estimate (with serious limitations discussed at the end of the derivation) of the resulting *B*^HFPC^ can then be easily computed as follows.

We consider the charge density

53

which is zero outside a sphere with radius *R* (centered at the origin **r** = **0**) and inside that sphere has a uniform gradient of magnitude |∇ρ(**r**)| = Γ in the *z*-direction, and

54

The condition ρ(**r**) ≥ 0 implies

55

The Hartree potential (inside the sphere) and the Hartree energy are given by^21^

56

57

To find the minimizing position **r**_min_ ≡ (*x*_min_, *y*_min_, *z*_min_) of the potential – *v*_H_(*r*^2^, *z*), we write *r̅* = *r*/*R*, *z̅* = *z*/*R*,

58

Setting here the partial derivatives (with respect to *x̅*, *y̅*, *z̅*) to zero, we obtain

59

The electron now sits at the position **r**_min_ = (0, 0, *z*_min_) . Setting in eq 58*r̅* = *z̅* = *z̅*_min_ (and expanding around κ = 0), then adding *U*[ρ] to obtain *E*_el_[ρ], yields

60

61

Replacing the cell radius *R* with the local Seitz radius *r*_*s*_(**r**), this result should be compared with the PC DFT one in eq 22 of ref (21),

62

We see that this simple model correctly captures the sign of *B*^HF^, which is negative, contrary to the positive sign for the DFT case. The order of magnitude is also correct, as eq 61 yields *B*^HFPC^ =  = −0.0598195. However, comparison with the results of section 3 shows that this *B*^HFPC^ is too large in magnitude, by almost a factor 4. We believe that the reasons for this discrepancy are (i) the fact that the PC cells with their electron have now a dipole moment invalidates the hypothesis that the energy can be computed as a sum of the energies of weakly interacting cells. The total energy is probably raised due to the cell–cell interaction that might also alter the position of the electron and the size of the PC cell; (ii) similarly to the exchange functional of DFT,^23^ the GEA2 coefficient obtained from a uniform system weakly perturbed is probably not equal to the result obtained for neutral atom densities.

## Accession Codes

All the data for *E*_el_[ρ] and *W*_1/2_^HF^[ρ], including the minimizing positions are available on zenodo at htttp://10.5281/zenodo.5734771.
